# De Novo Assembly and Comparative Analysis of Mitochondrial Genomes of Two *Pueraria montana* Varieties

**DOI:** 10.3390/ijms25115656

**Published:** 2024-05-22

**Authors:** Lijun Guo, Guoren Lao, Longfei He, Dong Xiao, Jie Zhan, Aiqin Wang

**Affiliations:** 1 National Demonstration Center for Experimental Plant Science Education, College of Agriculture, Guangxi University, Nanning 530004, China; 2117401014@st.gxu.edu.cn (L.G.); 2117301018@st.gxu.edu.cn (G.L.); xiaodong@gxu.edu.cn (D.X.); may2399@163.com (J.Z.); 2 Agricultural and Animal Husbandry Industry Development Research Institute, Guangxi University, Nanning 530004, China; lfhe@gxu.edu.cn; 3 Key Laboratory for Agro-Environment and Agro-Product Safety, Guangxi University, Nanning 530004, China; 4 Key Laboratory of Crop Cultivation and Tillage, Guangxi University, Nanning 530004, China

**Keywords:** *Pueraria montana*, mitochondrial genome, comparative analysis, RNA editing, synteny analysis

## Abstract

*Pueraria montana* is a species with important medicinal value and a complex genetic background. In this study, we sequenced and assembled the mitochondrial (mt) genomes of two varieties of *P. montana*. The mt genome lengths of *P. montana* var. *thomsonii* and *P. montana* var. *montana* were 457,390 bp and 456,731 bp, respectively. Both *P. montana* mitogenomes showed a multi-branched structure consisting of two circular molecules, with 56 genes annotated, comprising 33 protein-coding genes, 18 tRNA genes (*trnC-GCA* and *trnM-CAU* are multi-copy genes), and 3 rRNA genes. Then, 207 pairs of long repeats and 96 simple sequence repeats (SSRs) were detected in the mt genomes of *P. montana,* and 484 potential RNA-editing sites were found across the 33 mitochondrial protein-coding genes of each variety. Additionally, a syntenic sequence analysis showed a high collinearity between the two mt genomes. This work is the first to analyze the mt genomes of *P. montana.* It can provide information that can be used to analyze the structure of mt genomes of higher plants and provide a foundation for future comparative genomic studies and evolutionary biology research in related species.

## 1. Introduction

*Pueraria* represents a genus encompassing 15–20 legume species [1] indigenous to regions spanning south, east, and southeast Asia, as well as New Guinea and northern Australia [2]. Within this genus, the plants manifest as lianas, shrubs, or climbing herbs, often characterized by sizable tuberous roots. They thrive in various habitats, including seasonally dry tropical and subtropical forests, rainforests, forest margins, and scrub vegetation, with a predilection for limestone outcrops and rocky terrains [1]. Notably, *P. montana* is indigenous to east Asia and has been translocated to numerous other countries for ornamental shading, high-nitrogen forage cultivation, soil erosion control, and material manufacturing [3]. In some countries within its native range, its roots serve as a starch source for traditional cuisine and medicine [4], while its stalk fiber has applications in textile production and fishing net construction [5].

The divergence of *P. montana* from its closely related species is estimated to have occurred approximately 4.63 million years ago. This species has been classified into three varieties: var. *lobata*, var. *montana*, and var. *thomsonii*. Nevertheless, taxonomic features such as the size of the flowers and the shape of the terminal leaflets exhibit overlap among these varieties [6]. For instance, the pedicel lengths range from 2 to 6 mm, 3 to 7 mm, and 1.5 to 4 mm, respectively. Particularly noteworthy is the intermediate morphology observed in the quantitative traits (e.g., the width and length of apex leaflets and the length of the pedicel) between *P. montana* var. *thomsonii* and *P. montana* var. *lobata*, suggesting that classifying and ranking *P. montana* solely based on morphological characteristics may be a questionable practice. Consequently, these three varieties pose considerable challenges for identification based on their morphological features.

Various molecular markers have been employed to elucidate the evolutionary relationships within the *Pueraria* genus and among legume species. However, the exact phylogenetic positioning of *P. montana* remains contentious because of their morphological similarities and the utilization of different molecular markers [7,8,9]. Previous studies have presented conflicting results: in some, *P. montana* var. *lobata* and the other two varieties were grouped within a clade nested within *P.* sensu stricto, as indicated by two cpDNA markers, while forming a polytomy when assessed using a nuclear marker (AS2) [10]. In another study, *Pueraria montana* var. *montana* was suggested to be a single species, with *P. montana* var. *thomsonii* being a sister to *P. montana* var. *lobata* based on nrITS sequences involving four accessions of *P. montana* and its varieties [11]. However, the relationships among these taxa were not definitively resolved. One study used 47 chloroplast genomes, which included the publicly accessible genomes of Pueraria and other legumes, to create phylogenetic trees. This included 7 *P. montana* var. *lobata*, 14 *P. montana* var. *thomsonii*, and 6 *P. montana* var. *montana*. The study showed that *P. montana* var. *lobata* and *P. montana* var. *thomsonii* grouped together, forming a clade, whereas all samples of *P. montana* var. *montana* formed a separate unique cluster using cp genomes [9].

Mitochondria, vital organelles in plant physiology and development, fulfill crucial roles in energy conversion, biosynthesis, and signal transduction [12]. Moreover, they exhibit extensive horizontal gene transfer (HGT) and RNA-editing mechanisms, ensuring functional integrity and a stable gene expression [13,14]. In most higher plants, chloroplasts and mitochondria are inherited maternally, a genetic phenomenon that diminishes paternal lineage influence, thereby facilitating genetic research [15]. Mitogenomes, characterized by lower evolutionary rates than plastid genomes, offer a suitable avenue for exploring phylogenetic relationships at the family, order, or higher taxonomic levels. Seed plants reportedly exhibit a mutation rate ratio of approximately 1:3:10 for mitochondrial, chloroplast, and nuclear genes, respectively [16]. As sequencing technology advances, mitogenomes are increasingly being analyzed to unravel their taxonomic classification and evolutionary dynamics [17]. However, to date, only one complete mt genome of *P. montana* has been deposited in GenBank.

This study aimed to characterize the mitogenomes of two *P. montana* varieties, sequencing and assembling their complete mitogenomes using Illumina and Nanopore technologies. The objectives were as follows: (1) elucidate the molecular features of *P. montana* mitogenomes and (2) enhance the understanding of *P. montana* organelle genome evolution through computational analyses of GC content, codon usage preference, repeats, RNA editing site prediction, and synteny.

## 2. Results

### 2.1. Mitochondrial Genome Assembly and Annotation

We used the Illumina and Nanopore sequencing systems to sequence DNA samples of *P. montana* var. *thomsonii* and *P. montana* var. *montana* in order to gather basic data for mitogenome assembly. For each of the branch points in the assembly that could potentially represent assembly artifacts, we exported the sequences around these branch points and mapped them back to the original long-reads. In cases where multiple potential connections existed at a branch point, we prioritized those connections that were supported by a higher number of long-reads. Our analysis revealed that contig 1 (ctg1) could form circular DNA molecules with both contig 2 (ctg2) and contig 3 (ctg3). Following the resolution of branch points based on the long-read data, we obtained two circular molecules (Molecule 1 and Molecule 2), and the sequences of both molecules are shown in Supplementary File S1. To prevent unnecessary calculations of ctg1 in the two circular DNA sequences, we manually removed the redundant ctg1 from the ctg1–ctg2 circular sequence, keeping only the ctg1–ctg2–ctg3 sequence for further analysis. Two newly sequenced complete mitogenomes were deposited to GenBank with the following Accession Numbers: *P. montana* var. *thomsonii* (PP275071 and PP275072) and *P. montana* var. *montana* (PP275073 and PP275074).

**Figure 1:**
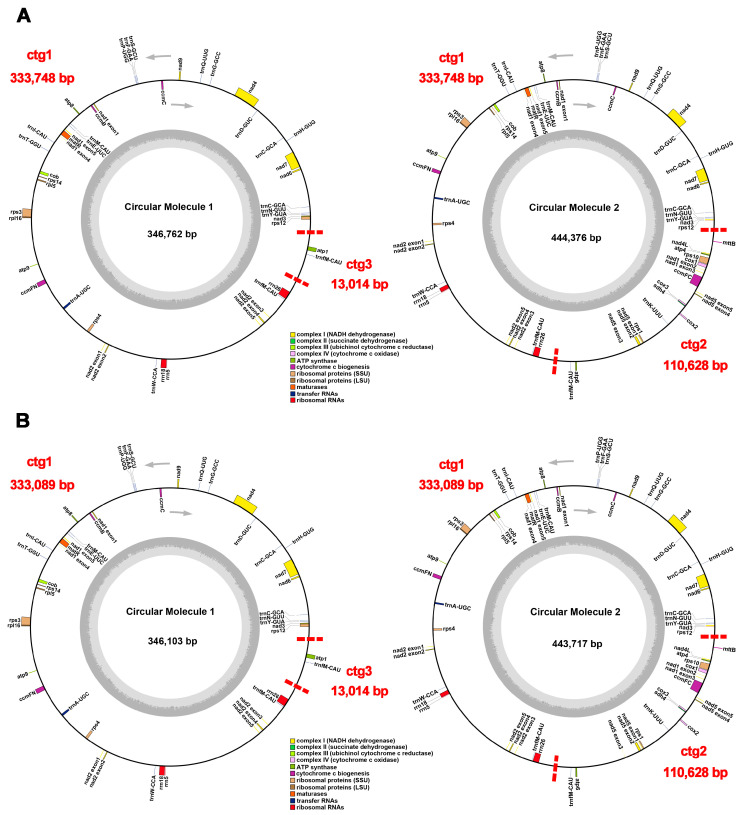
Structure and characteristics of the complete mitochondrial genomes of *P. montana* var. *thomsonii* (**A**) and *P. montana* var. *montana* (**B**). The arrows indicate that genes inside the circle are transcribed in the clockwise direction, and genes outside the circle are transcribed in the counter-clockwise direction. Different colors indicate genes belonging to different functional groups. Dark gray in the inner circle indicates the GC content, and light gray indicates AT.

The sizes of the *P. montana* var. *thomsonii* and *P. montana* var. *montana* mt genomes were 457,390 bp and 456,731 bp, respectively. This discrepancy in size was attributed to ctg1, which exhibited a 659 bp difference between the two cultivars (Figure 1). The size of the PCGs was 30,234 bp, taking up 81.73% of the mt genes’ length (Table 1). The lengths of the rRNAs and tRNAs were 2589 bp and 1561 bp, respectively. The mitogenome contained a total GC content of 45.05%, composed of 42.91% PCGs, 51.75% rRNAs, and 51.78% tRNAs (Table 1). The GC content was the lowest in the PCGs compared to the rRNAs and tRNAs.

In both mt genomes, 56 genes were annotated, consisting of 33 PCGs, 18 tRNA genes (*trnC-GCA* and *trnM-CAU* are multi-copy genes), and 3 rRNA genes (Table 1). The PCGs included eight main categories of core genes: five ATP synthase genes (*atp1*, *atp4*, *atp6*, *atp8*, and *atp9*), nine NADH dehydrogenase genes (*nad1*, *nad2*, *nad3*, *nad4*, *nad4L*, *nad5*, *nad6*, *nad7*, and *nad9*), four ubiquinol cytochrome c reductase genes (*ccmB*, *ccmC*, *ccmFc*, and *ccmFn*), three cytochrome C oxidase genes (*cox1*, *cox2*, and *cox3*), one maturation enzyme gene (*matR*), one panthenol-cytochrome C reductase gene (*cytb*), one succinate dehydrogenase (*sdh4*), and one membrane transport protein gene (*mttB*). The non-core genes consisted of two ribosomal large subunit genes (*rpl5* and *rpl10*) and six ribosomal small subunit genes (*rps1*, *rps3*, *rps4*, *rps10*, *rps12*, and *rps14*). 

### 2.2. Codon Preference of the Mitogenome

**Figure 2:**
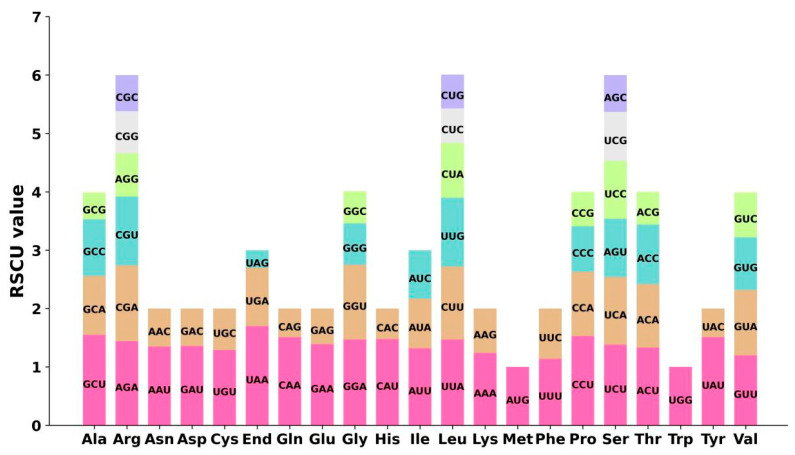
Relative synonymous codon usage (RSCU) of mitochondrial genomes of *P. montana* var. *thomsonii*.

The codon usage bias of the 33 unique PCGs in the mt genomes of the two *P. montana* varieties was analyzed, respectively, and the utilization of each amino acid for codons is detailed in Table S1. The codon preference was comparable across the two cultivars, with just *P. montana* var. *thomsonii* being used as an example (Figure 2). Serine (Ser), Arginine (Arg), and Leucine (Leu) were the most often used amino acids, whilst methionine (Met) and tryptophan (Trp) were the least utilized amino acids among the 33 PCGs. Codons with a Relative Synonymous Codon Usage (RSCU) over one are thought to be favored by amino acids. Apart from the initiation codon (AUG) and the tryptophan codon (UGG), both of which had an RSCU value of one, the mitochondrial PCGs exhibited a strong tendency for certain codons. For instance, the termination codon for UAA showed the greatest preference among the mitochondrial PCGs, with an RSCU value of 1.7 (Figure 2). Additionally, alanine (Ala) preferred the codon GCU, with an RSCU value of 1.55. These data results reveal a substantial tendency toward a high proportion of NNA and NNU, comparable to other terrestrial plant species [18].

### 2.3. Repeat Elements

**Figure 3:**
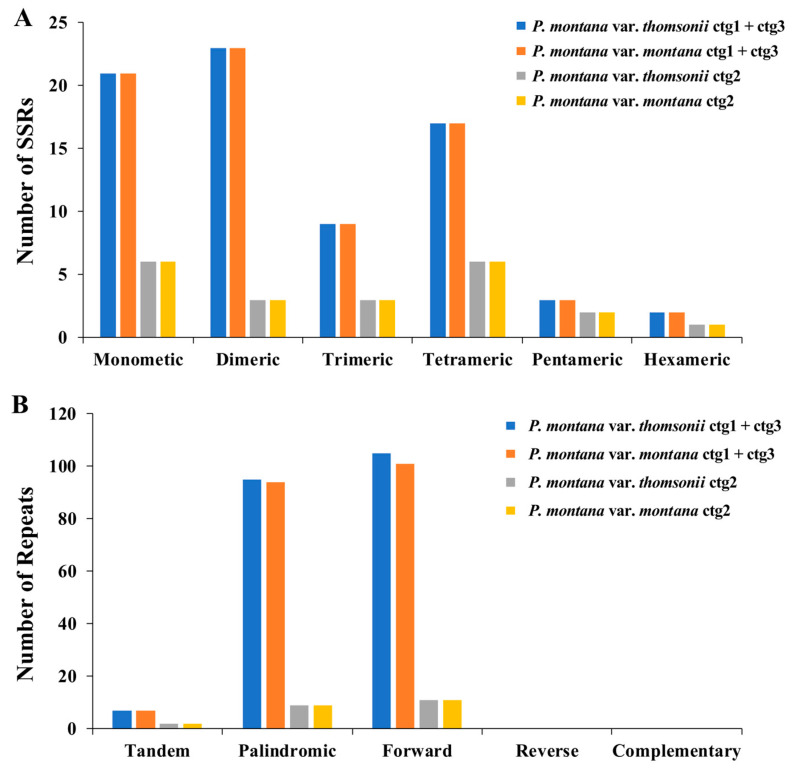
Number of repeat elements in mt genomes of *P. montana* var. *thomsonii* and *P. montana* var. *montana.* (**A**) Number of SSRs, SSR, simple sequence repeat. (**B**) Number of repeated sequences.

Inheritance of the cp genome is uniparental, and within a given species, there exists a notable degree of variation in simple sequence repeats (SSRs) [19]. In ctg1 + ctg3 and ctg2, we identified 75 and 21 SSRs, respectively (Figure 3). In ctg1 + ctg3, monomeric and dimeric forms constituted 58.67% of the total SSRs, with thymine (T) monomer repeat sequences comprising 52.38% (11 out of 21) of the monomer SSRs. The most abundant forms of SSRs in ctg2 were monomeric and tetrameric, accounting for 57.14% (12) of the total SSRs. Adenine (A) monomer repeat sequences represented 66.67% (four out of six) of the monomer SSRs. Among the two mitochondrial molecules, TA repeat sequences were the most common type of dimeric SSRs, accounting for 30.77 and 25.00% of the dimeric SSRs, respectively.

In biological cell DNA sequences, there are a vast number of long repeats, such as tandem repeats (TRs) and interspersed repeats, which are categorized based on size as large (LR, >500 bp), medium (IntR, 50–500 bp), and short (SR, <50 bp) repeats [20]. A mitogenome analysis showed that ctg1 + ctg3 harbored seven TR sequences with a match rate exceeding 81% and lengths ranging from 13 to 96 bp. A total of 200 pairs of interspersed repeats with a length of 30 or more were found, consisting of 95 palindromic repeats and 105 forward repeats identified. There were no detectable reverse repeats or complementary repeats. The longest palindromic repeat was 945 bp, whereas the longest forward repeat was 180 bp.

### 2.4. RNA-Editing Events

**Figure 4:**
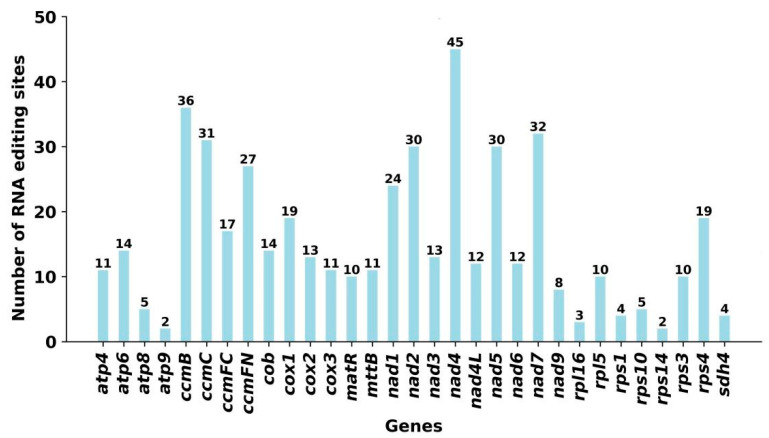
RNA-editing sites in different coding genes of *P. montana* var. *thomsonii*.

The RNA-editing events in the 33 unique PCGs in the mitogenomes of the two *P. montana* varieties were identified. A cutoff value of 0.9 was set as the standard. Using this criterion, 484 potential RNA-editing sites were found across the 33 mitochondrial PCGs. Both varieties exhibited identical RNA-editing sites; hence, only one is shown in Figure 4. Editing sites in both samples were only identified at the first and second positions of the triplet codes, with no occurrences at the third position (Table S2). Furthermore, all the editing sites were involved in converting the base C into U. Among the mitochondrial genes, the *nad4* gene had the largest number of RNA-editing sites, with 45 identified sites, making it the most extensively edited gene. Following *nad4*, the *ccmB* gene experienced the second-highest frequency of RNA-editing events, with 36 occurrences among all mitochondrial genes. In order to evaluate the precision of this prediction, a random selection of 22 genes was made, which included a total of 374 predicted RNA-editing sites. The technique combining PCR amplification and Sanger sequencing was used. Among them, 321 RNA sites were successfully verified (Figure S1 and Supplementary File S2).

### 2.5. Synteny Analysis

**Figure 5:**
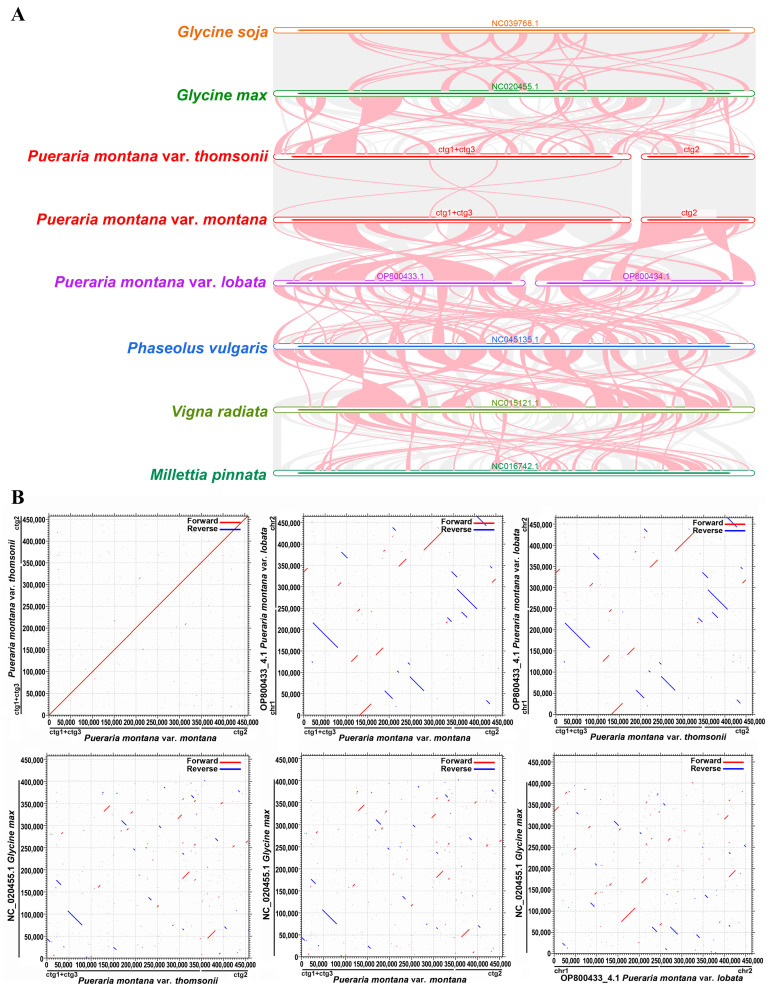
Collinearity analysis (**A**) and dot plot (**B**) of the mitochondrial genome of *P. montana* species with close relatives. (**A**) the red arc area implies inversion, whereas the gray area represents areas with high homology. (**B**) Red lines in the boxes represent forward comparisons, and blue lines represent reverse complementary comparisons.

A co-linearity analysis was used to investigate the evolutionary patterns across the species by evaluating homologous genes or sequence alignments. The collinearity analysis of the *P. montana* mt genome is shown in Figure 5A. There were many collinear blocks between the three *P. montana* cultivars, most of which were more than 90% homologous. The longest homologous sequence between *P. montana* var. *thomsonii* and *P. montana* var. *lobata* was approximately 58,634 bp, with a mismatch of 18 bp. There were smaller collinear blocks between *P. montana* cultivars and *Glycine max*. That is, the longest homologous sequence was 32,789 bp, with a homologous rate of 98.69%.

Homologous collinear blocks were identified between *P. montana* varieties and *Glycine max* within the Fabaceae order in a dot plot (Figure 5B). These results demonstrate significant similarity in the sequencing of the mitochondrial genomes between the *P. montana* var. *thomsonii* and *P. montana* var. *montana* samples, with only minor changes in the organization of the genome. This is similar to that seen between the *Glycine soja* and *G. max* samples. Conversely, the mitogenomes of different species exhibited extensive rearrangements and lacked structural conservation.

### 2.6. Substitution Rates of PCGs

**Figure 6:**
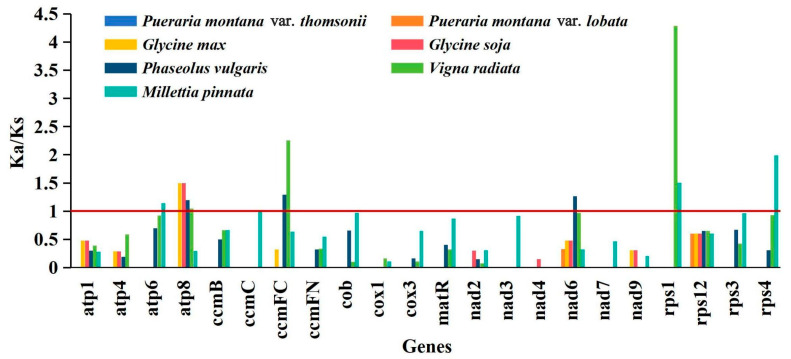
Ka/Ks values of *P. montana* species with close relatives.

Calculating non-synonymous substitutions (Ka) and synonymous substitutions (Ks) is crucial for reconstructing phylogeny and understanding the evolutionary processes of protein-coding regions in closely related species [21]. The Ka/Ks ratio in genetics is used to assess the presence of selection pressure on a particular protein-coding gene during evolution. When Ka/Ks is more than one, it indicates positive selection. When Ka/Ks equals one, it indicates neutral selection. When Ka/Ks is less than one, it indicates negative selection [22]. The 27 PCGs from the *P. montana* var. *montana* mt genome were compared with those of 7 samples from 6 species, *P. montana* var. *thomsonii* (PP275071 and PP275072), *P. montana* var. *lobata* (OP800433.1 and OP800434.1), *Glycine max* (NC_020455.1), *G. soja* (NC_039768.1), *Phaseolus vulgaris* (NC_045135.1), *Vigna radiata* (NC_015121.1), and *Millettia pinnata* (NC_016742.1) for the Ka/Ks calculation, excluding results for 5 PCGs that exhibited Ka/Ks values of zero across all compared species (Figure 6). Overall, no Ka/Ks values higher than one were observed in *P. montana* var. *montana* compared to *P. montana* var. *thomsonii* and *P. montana* var. *lobata*, suggesting that mt genes are highly conserved among three varieties of *P. montana*. The Ka/Ks values of *P. montana* var. *montana atp8* compared to *G. max* and *G. soja* suggest that positive selection occurred during evolution between *Pueraria* and *Glycine*. Most genes (21) experienced negative selection throughout their evolution, as shown by the Ka/Ks values, which made up 77.78% of the 27 protein-coding genes, being less than one when compared to other plant species. These findings indicate that mitochondrial genes are well-preserved over the evolutionary process in Fabaceae plants.

## 3. Discussion

Mitochondria serve as the powerhouses of plants, generating the energy necessary for life processes. Compared to animals, plant mitochondria exhibit greater genomic complexity, encompassing size variations, repeat content, sequence arrangements, and a highly conserved coding sequence [23]. In this study, we sequenced and assembled two mt genomes of *P. montana* and performed a comprehensive comparative analysis comparing the two mt genomes. Studying the mt genome sequences of two *P. montana* varieties can biologically increase the understanding of the evolution of *Pueraria* species.

The size of plant mitogenomes is notably larger compared to other species. For instance, animal mitogenomes typically range from 15 to 17 kb [24], while algal mitochondria, slightly larger, can span from 13 to 96 kb. In contrast, angiosperm mitogenomes typically fall within the 200 to 700 kb range [20]. Here, we observed that the mitogenome size of *P. montana* reached 457 kb. However, the coding regions in the mitochondrial genome accounted for 8.08% of the overall genome, consistent with prior findings in angiosperms, where coding portions usually account for 7–17% of the total mt genome, with intergenic regions occupying the remainder [25]. Plant mitogenomes typically harbor a relatively small number of coding genes, usually ranging from 50 to 60 [26]. With 55 genes annotated, including 33 PCGs, in the *P. montana* mitogenome, the gene count closely resembled *Glycine max* [27]. The GC content serves as another important indicator for species assessment. In the *P. montana* mitogenome, the GC content was determined to be 45.05%, akin to the GC content observed in the mitogenomes of *G. max* (44.90%) [27], and higher than that of the *P. montana* chloroplast genome (34.00%) [9]. Notably, the *P. montana* mitogenome exhibited a multi-branched structure, comprising two circular molecules. While most published plant mitogenomes are depicted as one circular DNA molecule, there are also alternative conformations, such as Y- and H-type linear forms. It is important to note that mitochondrial DNA, in its natural state, often comprises a mixture of various conformational forms [26].

The mitogenomes of higher plants exhibit distinctive expression patterns, including RNA editing and codon preference [28]. Codon usage bias is the uneven frequency of synonymous codon usage in coding DNA [29], meaning that synonymous codons are not uniformly utilized in gene transcripts to encode all amino acids, except for methionine (Met) and tryptophan (Trp) [30]. Codon usage bias is a widespread phenomenon in the natural world and represents molecular evolution [31]. An analysis of codon usage preferences in the *P. montana* mitogenome revealed that, similar to most other plants, leucine (Leu), serine (Ser), and arginine (Arg) were the most frequently occurring amino acids. At the same time, Met, cysteine (Cys), and Trp were much less common [32]. The preference for codons ending in A/T in genes encoded in the *P. montana* mitogenome was consistent with the codon preferences observed in most angiosperms [33,34]. However, compared with the codon preference in chloroplast genomes, codon usage bias analyses of mitogenomes, especially in plant mitogenomes, are relatively rare. This scarcity may be attributed to the lower number of plant mitogenomes sequenced compared to chloroplast genomes.

One notable characteristic of higher plant mitogenomes is their prevalence of repeats. The presence of numerous repeats contributes to frequent homologous recombination events and increases susceptibility to gene rearrangements, which is closely associated with the complexity of mitochondrial gene structure [35]. In this study, we extensively examined SSRs and long TRs, as well as non-tandem repeats. Both varieties of the *P. montana* mitogenome contained ample repeat sequences, suggesting that intermolecular recombination frequently occurs in the mitochondrial genome. This dynamic process results in sequence and conformational changes during evolution.

Numerous previous studies have highlighted RNA editing as a crucial process for gene expression in both the mitochondrial and chloroplast genomes of higher plants [36,37]. Out of the 484 RNA-editing sites found in our analysis, none were situated in the third position. This lack of expected RNA-editing sites at inactive places was probably due to the constraints of the PREP-Mt prediction approach rather than the absence of RNA editing in such positions. Since the majority of RNA-editing sites at third-codon positions do not change the amino acid, the decision criteria used by PREP-Mt may not be able to choose the altered version [38]. RNA-editing events can lead to changes in initiation and termination codons in PCGs, with the frequency of their occurrence surpassing that of their removal. We observed that the *nad1* and *nad4L* genes utilized ACG as initiation codons, which may be modified to the normal AUG through RNA editing. Furthermore, the *ccmFC* gene and *rps10* employed CGA as termination codons, which may be altered to UGA via RNA editing, consistent with previous findings in other higher plants [18]. Interestingly, both *P. montana* varieties exhibited identical RNA-editing sites in our study. This observation aligns with earlier research indicating the lineage-specific nature of RNA editing, with variations in frequency and type occurring in each organelle [18,36].

A Ka/Ks analysis and comparison of mitogenome features offer a comprehensive insight into plant mitogenome evolution. Here, we found that *nad6*, *rps1*, *atp6*, *atp8*, *ccmFc*, and *rps4* underwent positive selection during evolution. Similar findings have been reported in various plant species, where genes such as *atp8*, *ccmFc*, *nad6*, *atp9*, *matR*, *ccmB*, and *mttB* were found to be under positive selection pressure during evolution [17,33,39]. The *P. montana* mitogenome demonstrates conservation, with most PCGs undergoing neutral or negative selection compared to other Fabaceae species. Overall, our investigation aligns with previous reports [18]. Additionally, we observed Ka/Ks values over 1.0 for *atp8* when comparing the *P. montana* mitogenome with those of four other Fabaceae species, indicating that this gene may have undergone positive selection during the evolution of Fabaceae. The analysis of transition to transversion ratios in our study provides insights into the evolutionary pressures on *P. montana* mitochondrial genomes. However, the interpretation of these findings is constrained by the relatively small sample size of eight genomes. Future research including a wider range of *P. montana* genomes from various subspecies and closely related species will provide more reliable mutation rates of selection sites.

## 4. Materials and Methods

### 4.1. Plant Materials and Sequencing

The leaves of well-grown living *P. montana* var. *thomsonii* and *P. montana* var. *montana* were collected from the plant cultivation research base of the College of Agriculture, Guangxi University, in Nanning, Guangxi (108°33′45″ E, 22°82′13″ N), China. The total DNA was acquired using the procedures outlined in the Tiangen TIANamp Genoic DNA Kit for blood/cell/tissue genomic DNA extraction (Tiangen Biotech, Beijing, Co., Ltd., Bejing, China).

The Illumina Novaseq 6000 platform and the Nanopore GridION sequencing platform (Oxford Nanopore Technology, Oxford Science Park, Oxford, UK) were employed for sequencing and library preparation, resulting in the acquisition of raw sequence data. Subsequently, Trimmomatic was utilized to obtain clean data [40]. In this step, we removed low-quality sequences from the Illumina dataset, specifically those with a quality value of Q ≤ 20 and sequences containing more than 5% of bases identified as “N”. We also eliminated low-quality sequences obtained by Nanopore sequencing, which included sequences with a quality value below Q < 7 [41], comprising around 1.8% of the total bases (Figure S2).

### 4.2. Genome Assembly and Annotation

Firstly, based on the long-read data obtained by Nanopore sequencing, the mitochondrial genomes of *P. montana* var. *thomsonii* and *P. montana* var. *montana* were assembled. Flye software (v2.9.2-b1786) [42] was employed with default parameters for the direct assembly of long-read sequencing data, yielding a graphical assembly result in GFA format. Subsequently, we utilized makeblastdb to create a database for all the assembled fasta contigs. Using the BLASTn program with *Arabidopsis* mitochondrial genes as query sequences, contig fragments containing mitochondrial genome segments were found (parameters: “-evalue 1e-5 -outfmt 6 -max_hsps 10 -word_size 7 -task blastn-short”). Bandage software (v0.8.1) [43] was used to visualize the GFA files, and mitochondrial contigs were filtered based on BLASTn results, resulting in a draft of the *P. montana* mitochondrial genome. Then, BWA software (v0.7.17) [44] was utilized to align both the long-read and short-read data to the mitochondrial contigs, and aligned mitochondrial reads were filtered and exported for further assembly. Finally, by integrating the aforementioned short-read and long-read sequencing data obtained by Illumina sequencing and Nanopore sequencing, respectively, a hybrid assembly approach was employed to assemble the *P. montana* mitochondrial genome. Unicycler software (v0.4.8) [45] was employed with default parameters for hybrid assembly, resulting in the final *P. montana* mitochondrial genome. Visualization of the mitogenome was performed using Bandage software (v0.8.1) [43].

The PCGs of the mitochondrial genome were selected for annotation using *Arabidopsis thaliana* (NC_037304.1) and *Liriodendron tulipifera* (NC_021152.1) as reference genomes. Annotation was performed using Geseq software (v2.03) [46]. Additionally, the mitochondrial genome annotation tool IPMGA (http://www.1kmpg.cn/ipmga/, accessed on 5 December 2023) was used, specifically noted for its effectiveness in annotating splice sites and trans-splicing genes in angiosperms. The annotation of mitochondrial tRNA genes was conducted using tRNAscan-SE software (v2.0.11) [47], while rRNA genes were annotated using BLASTN software (v2.13.0) [48]. Any annotation errors in the mitochondrial genome were manually corrected by Apollo software (v1.11.8) [49].

### 4.3. RNA-Editing Prediction

We utilized the sequences of 33 unique PCGs from the mitogenomes as input files. We employed Deepred-mt [50] to forecast the C to U RNA-editing sites within the mitochondrial PCGs. Utilizing a convolutional neural network (CNN) model, this tool presents higher accuracy levels than its predecessors. All results exceeding a probability threshold of 0.9 were retained. For experimental verification, a random selection of 22 genes was made to confirm the RNA-editing sites. Primer sequences were created on both sides of the selected genes (Table S3). Complementary DNA (cDNA), which was generated from RNA using random primers, was used as a template for amplification. After amplification, the products were then analyzed using Sanger sequencing. Ultimately, by comparing the sequences of the products derived from the cDNA and mitogenome, we were able to ascertain the presence of RNA-editing processes.

### 4.4. Codon Usage Bias and Repeat Analysis

We employed MISA (v2.1) [51] and TRF (v4.09) [52] to identify repetitive sequences, comprising microsatellite sequence repeats, tandem repeats, and interspersed repeats. Subsequently, the REPuter web server (https://bibiserv.cebitec.uni-bielefeld.de/reputer/, accessed on 29 December 2023) [53] was utilized. The data were visualized by Excel software (2021) and the Circos package (v0.69-9) [54]. Phylosuite software (v1.1.16) [55] was utilized to extract the protein-coding sequence of the genome. Finally, Mega software (v7.0) [56] was employed to conduct a codon preference analysis on the PCGs of the mitochondrial genome and calculate the RSCU values.

### 4.5. Synteny Analysis

Using the BLAST program, pairwise comparisons of various mitochondrial genomes were conducted, and BLASTN results were obtained. Sequences with lengths exceeding 500 bp, indicating homology, were retained to create conservative collinear blocks for constructing a Multiple Synteny Plot. Based on sequence similarity, the original code of MCscanX [57] was employed to generate a dot plot comparing *P. montana* with *Glycine max*.

### 4.6. Non-Synonymous (Ka)/Synonymous (Ks) Analysis

The Ks/Ka substitution rates of the PCGs in the *P. montana* var. *montana* mitogenome were analyzed using seven related species as references. Sequence alignment was conducted using Mega 7.0, while Ka/Ks calculations were performed using DNAsP v6.12.

## 5. Conclusions

*Pueraria montana* is a species with important medicinal value and a complex genetic background. Our comprehensive analysis of the *P. montana* mitogenomes successfully highlighted the mitogenomes of two *P. montana* samples in the form of a multi-branched structure consisting of two circular molecules. 

Both *P. montana* varieties shared the same number of SSRs, with 96 SSRs in total. We identified 484 potential RNA-editing sites across 33 mitochondrial PCGs, of which the *nad4* gene had the largest number of RNA-editing sites. An evolutionary analysis revealed that most PCGs in different genera indicated negative selection, while *atp8* in *Pueraria* and *Glycine* indicated the existence of positive selection. These findings provide a foundation for future comparative genomic studies and evolutionary biology research on related species.

## Figures and Tables

**Table 1:** Genome features of the *Pueraria montana* mt genomes.

Genome Feature	*P. montana* var. *thomsonii*	*P. montana* var. *montana*
Genome size (bp)	457,390	456,731
Numbers of contigs	2	2
Contig length(bp)	Molecule 1: 346,762 Molecule 2: 444,376	Molecule 1: 346,103 Molecule 2: 443,717
GenBank Nos	ctg1 + ctg3: PP275071 ctg2: PP275072	ctg1 + ctg3: PP275073 ctg2: PP275074
GC content (%)	45.05	45.05
Length of the protein-coding region (bp)	30,234	30,234
GC content of the protein-coding region (%)	42.91	42.91
Length of rRNA genes (bp)	5272	5272
GC content of rRNA genes (%)	51.75	51.75
Length of tRNA genes (bp)	1487	1487
GC content of tRNA genes (%)	51.78	51.78
Number of protein-coding genes (native)	33	33
Number of protein-coding genes (plastid-derived)	2	2
Number of rRNA genes	3	3
Number of tRNA genes (native)	13	13
Number of tRNA genes (plastid-derived)	5	5
Total genes	56	56

## Data Availability

All data cited in the study are publicly available.

## Supplementary Materials

The supporting information can be downloaded at: https://www.mdpi.com/article/10.3390/ijms25115656/s1.
