# Supplementary material for: De Novo Assembly and Comparative Analysis of Mitochondrial Genomes of Two Pueraria montana Varieties
Source: Int J Mol Sci. 2024 May 22;25(11):5656. doi: 10.3390/ijms25115656 (PMC11171644; doi:10.3390/ijms25115656)
Supplement: Supplementary file 1 [file ijms-25-05656-s001.zip › ijms-2928843 supplementary/Figure S1 and Figure S2.doc]

**Figure S1.** Electrophoresis plots of PCR products for the four editing sites (A) and sequencing editing site comparison of CDS and cDNA (B). M denotes Maker 5000, N denotes negative control, 1, 2, 3, and 4 denote *cox2* (762bp), *nad9* (598bp), *rps10* (413bp), and *rps14* (303bp), respectively.

**Figure S2.** Distribution of quality scores across all reads of Nanopore data
